# Fungal Community Associated with *Dactylopius* (Hemiptera: Coccoidea: Dactylopiidae) and Its Role in Uric Acid Metabolism

**DOI:** 10.3389/fmicb.2016.00954

**Published:** 2016-06-23

**Authors:** Arturo Vera-Ponce de León, Alejandro Sanchez-Flores, Mónica Rosenblueth, Esperanza Martínez-Romero

**Affiliations:** ^1^Programa de Ecología Genómica, Centro de Ciencias Genómicas, Universidad Nacional Autónoma de MéxicoCuernavca, Mexico; ^2^Unidad de Secuenciación Masiva y Bioinformática, Instituto de Biotecnología, Universidad Nacional Autónoma de MéxicoCuernavca, Mexico

**Keywords:** fungal-metagenomics, *Cryptococcus*, scale insects, *Rhodotorula*, ITS region, purine metabolism, carmine cochineal

## Abstract

We studied fungal species associated with the carmine cochineal *Dactylopius coccus* and other non-domesticated *Dactylopius* species using culture-dependent and -independent methods. Thirty seven fungi were isolated in various culture media from insect males and females from different developmental stages and *Dactylopius* species. 26S rRNA genes and ITS sequences, from cultured fungal isolates revealed different species of *Cryptococcus, Rhodotorula, Debaryomyces, Trametes*, and *Penicillium*, which are genera newly associated with *Dactylopius*. Uric acid (UA) and uricase activity were detected in tissues extracts from different insect developmental stages. However, accumulation of high UA levels and low uricase activities were found only after antifungal treatments, suggesting an important role of fungal species in its metabolism. Additionally, uricolytic fungal isolates were identified and characterized that presumably are involved in nitrogen recycling metabolism. After metagenomic analyses from *D. coccus* gut and hemolymph DNA and from two published data sets, we confirmed the presence of fungal genes involved in UA catabolism, suggesting that fungi help in the nitrogen recycling process in *Dactylopius* by uricolysis. All these results show the importance of fungal communities in scale insects such as *Dactylopius*.

## Introduction

Insects are the most diverse arthropods in the biosphere and dwell in almost all environments. They can feed on a wide variety of nutrients, probably due to their associated microorganisms, including fungal species (Douglas, 2009). There is evidence that many arthropods harbor yeast-like microorganisms inside their bodies (Buchner, 1965), and at least eight orders of insects, including 143 species, have been reported to be associated with fungi (Vega and Blackwell, 2005; Gibson and Hunter, 2010). Fungi are located either inside the insect body in highly specialized cells called mycetocytes, as in *Nilaparvata lugens* and *Drosophila melanogaster*, which harbor yeasts (Chen et al., 1981; Ebbert et al., 2003), or in cavities named mycangia as in bark beetles (Jones et al., 1999; Klepzig and Six, 2004; Ganter, 2006). Fungi have also been found in the insect gut, as well as in their reproductive organs and fat tissues (Buchner, 1965; Gibson and Hunter, 2009; Rivera et al., 2009; Ricci et al., 2011). Moreover, studies on fungi-insect symbioses show that fungi play important roles in insect development and fitness (Gibson and Hunter, 2010). Fungi are capable of providing nitrogen compounds that are limited in the diets of some insects, or can degrade high molecular weight molecules and produce pheromones for mating and communication (Brand et al., 1976; Sasaki et al., 1996; Nasir and Noda, 2003; Gibson and Hunter, 2010). In some insects like cockroaches, termites, shield bugs, planthoppers, and bark beetles uric acid (UA), the major product of nitrogen excretion, is recycled by bacterial or fungal symbionts (Mullins and Cochran, 1975; Potrikus and Breznak, 1981; Pant, 1988; Kashima et al., 2006; Morales-Jiménez et al., 2013; Patiño-Navarrete et al., 2014). However, to our knowledge, there are no reports on the UA content or catabolism in scale insects.

The Dactylopiidae family includes only one genus, *Dactylopius* (Costa), commonly called “cactus cochineals” or “cochineal scale insects.” They are obligate phytophagous hemipterous from the scale insects family (Coccoidea). Ten species have been described as belonging to this genus and six of them, *D*. *ceylonicus, D*. *confusus, D*. *opuntiae, D*. *coccus, D. bassi*, and *D*. *tomentosus*, inhabit Mexico (Ben-Dov and Marotta, 2001; Chávez-Moreno et al., 2009). These insects are the main source of carminic acid, a glycoside-anthraquinone molecule used in the textile, cosmetic, pharmaceutical, and food industries as a dye or pigment (Deveoglu et al., 2011). All of these *Dactylopius* species produce carminic acid, but only *D. coccus* is cultivated and used for commercial purposes due to the higher amount and quality of its pigment (Rodríguez et al., 2005). Moreover, since non-cultivated *Dactylopius* are considered a cactus plague, in some countries they are used as biological control for these plants (Zimmermann and Moran, 1991; Spodek et al., 2013; Pérez-Ramirez et al., 2014; da Silva Santos et al., 2015).

*Dactylopius* cochineals spend their life feeding on *Opuntia* and *Nopalea* cactus sap (Chávez-Moreno et al., 2009), which is mainly composed of water (88–95% wet weight) and has low protein concentration (0.5–1% wet weight; Stintzing and Carle, 2005). Thus, we supposed that nitrogen deficiencies may be supplied by associated symbiotic microorganisms. The diversity of microbial symbionts in *Dactylopius* has been scarcely described. There are a few reports of the bacterial communities in *Dactylopius* species (Pankewitz et al., 2007; Ramírez-Puebla et al., 2010, 2015). However, there are no reports on the fungal community and their possible roles in association with this cochineal insect. The aim of this work was to identify and describe fungi from diverse stages and tissues of *Dactylopius* species, as well as to determine their role in uric acid catabolism in these insects.

## Materials and methods

### Insect sampling and identification

*Dactylopius coccus* samples were obtained from Campo Carmín Company (Table 1). Wild species of *Dactylopius* (*D. opuntiae* and *D. confusus*) were collected from three states in Mexico (Table 1). Insects were obtained from *Opuntia* spp. cactus and were transported together with their host plants to the laboratory. For species identification, ten female adults from the different locations were preserved in fixation buffer (chloroform: ethanol: glacial acetic acid 4:3:1). The superficial wax was removed by placing the insects in 10% KOH for 10 min at 60°C. Body contents were removed by cutting a slit in the body margin and expelling the contents with a spatula. Cleaned specimens were transferred into 70% alcohol for 10 min. Then, all specimens were transferred and kept in a staining solution (2% aqueous solution of acid fuchsin) overnight. Specimens were washed in 70% alcohol for 10 min and dehydrated in 100% alcohol for 10 min. Each specimen was placed face down on a slide with a drop of Canada balsam and covered with a slip. Microscopic observations with the keys described by Perez-Guerra and Kosztarab allowed the morphological identification of *Dactylopius* species (Perez-Guerra and Kosztarab, 1992). Specimens were deposited in the collection of Héctor González-Hernández from COLPOS, Mexico.

**Table 1:** **Collection sites of *Dactylopius* species**.

**Location**	**Location code**	**Latitude/Longitude**	**Insect species**
Campo Carmín, Xochitepec, Morelos state	CC	18°44′46.7″N	*D. coccus*
		99°11′17.8″W	
Teotihuacán, Mexico state	TEM	19°40′47.3″N	*D. opuntiae*
		98°50′59.4″W	
Ecatepec, Mexico state	ECM	19°35′27.3″N	*D. opuntiae*
		98°59′57.5″W	
Jiutepec, Morelos state	JM	18°53′52.5″N	*D. opuntiae*
		99°10′56.8″W	
Coyoacán, Federal district	CDF	19°19′18.9″N	*D. confusus*
		99°11′09.8″W	
Milpalta, Federal district	MADF	19°12′26.7″N	*D. confusus*
		99°1′28.8″W	

### Fungal isolation, DNA extraction, and PCR amplification

Insects from 1st instar nymph, 2nd instar nymph and adult stages of *D. coccus* and of wild *Dactylopius* (*D. opuntiae* and *D. confusus*) were detached from their host plant, submerged in 100% ethanol and the wax cover was removed with forceps under a stereoscope. They were then surface disinfected with 70% ethanol and rinsed twice with sterile water. A pool of five washed and disinfected insects from each developmental stage mentioned above of *D. coccus, D. opuntiae, D. confusus* and a pool of 20 *D. coccus* adult males were totally macerated (hereafter named as whole body samples) with a sterile Eppendorf® pestle in a 1.5 microtube with 500 μl of 0.85% NaCl. Additionally, two individuals of 2nd instar nymphs and adult females from *D. coccus, D. opuntiae*, and *D. confusus* were dissected under sterile conditions to obtain guts (gut samples) and ovary-eggs (ovary samples). Dissections were performed by making a transverse cut in the cuticle and removing the organs with fine sterile forceps. These organs were submerged in 600 μl of sterile 0.85% NaCl and macerated using sterile pestles. After maceration, all samples were indirectly sonicated for 30 s in a Bransonic® Ultrasonic MH Cleaning Bath. One hundred microliters of this suspension were inoculated in 50 ml of YPD media (1% w/v yeast extract, 2% w/v peptone, and 2% w/v dextrose), malt extract media (Difco) and two minimal media: MMT [NH_4_Cl 3 g l^−1^; K_2_HPO_4_ 1 g l^−1^; MgSO_4_ 0.025 g l^−1^; CaCl_2_ 0.25 g l^−1^; KCl 0.025 g l^−1^; FeSO_4_ 0.02 g l^−1^; yeast extract (Difco) 0.02 g l^−1^; trehalose 0.01 g l^−1^; glucose 10 g l^−1^; and sucrose 5 g l^−1^] and MMTC [NH_4_Cl3 g l^−1^; K_2_HPO_4_ 1 g l^−1^; MgSO_4_ 0.025 g l^−1^; CaCl_2_ 0.25 g l^−1^; KCl 0.025 g l^−1^; FeSO_4_ 0.02 g l^−1^; CuSO_4_ 0.02 g l^−1^; yeast extract (Difco) 0.02 g l^−1^; Carmine dye 0.01 g l^−1^ (Merck microscopy grade)] and were incubated at 25 ± 2°C at 180 rpm for 72 h. After the incubation period, 100 μl of the liquid medium was spread on the corresponding solid medium for selection of yeast and filamentous isolates. To test the best conditions for growing fungi, 100 μl of the initial macerate suspension was also spread directly on solid media MMTC and MMT and incubated in CO_2_ generation Gaspack™ EZ CampyPuch™ System at room temperature for 1 week. Pure cultures were obtained and stored at −70°C in 25% glycerol for further analysis.

DNA from fungal isolates was extracted following the protocols described by Hoffman and Winston (1987). ITS regions were amplified using primers ITS1 (5′ TCCGTAGGTGAA CCTGCGG 3′) and ITS2 (5′TCCTCCGCT TATTGATATGC 3′) that we designed for this study. D1-D2 26S rRNA gene region from fungal isolates were amplified using primers 26S-A1 (5′ CATATCAATAAGCGG AGCAAAAG 3′) and 26S-A2 (5′ìCAGTTCTGCTTACCAAAA ATGG 3′; Scorzetti et al., 2002). The final concentration for 50 μl PCR reactions was as follows: 10 ng of total DNA, 0.8 pmol of each primer, 0.2 mM dNTPs, 2.5 mM MgCl, 0.5 U *Taq* polymerase and 1x *Taq* polymerase buffer (Invitrogen Life Technologies, Sao Paulo, Brazil). The reaction conditions were 94°C for 5 min; 35 cycles of 60 s at 94°C, 60 s at 57°C, and 90 s at 72°C; and a final extension at 72°C for 10 min. PCR products were purified using the High Pure PCR Product Purification Kit (Roche) and sequenced by Macrogen Inc. (Seoul, Korea) by Sanger technology.

### Insect DNA extraction

For shotgun metagenomic analysis, 30 adult females of *D. coccus* were externally disinfected and dissected as described above. All 30 guts (including the Malpighian tubules) were placed in 200 μl of lysis buffer solution (Tris-HCl 10 mM, pH. 8; EDTA 1 mM; NaCl 10 mM; SDS 1%; Triton X-100 2%). For DNA extraction, samples were macerated with sterile pestles, additionally 0.3 g of sterile glass beads and 200 μl of phenol-chloroform-isoamyl alcohol (25:24:1) were added to the macerate. The samples were mixed by vortexing, warmed at 65°C for 1 h, followed by centrifugation at 15996 × g and the aqueous phase was recovered. Nucleic acids were precipitated with 1 ml of absolute ethanol for 20 min at −20°C, washed twice with 70% ethanol then dried in a vacuum concentrator, resuspended in 50 μl of deionized water and cleaned with DNeasy Blood and Tissue Kit (QIAGEN) columns (this sample is hereafter called as gut metagenome). Additionally, hemolymph from another 30 individuals of *D. coccus* adult females was obtained by dissection. Insect debris was separated by centrifugation in a Percoll (Sigma) gradient, and hemolymph cells were resuspended into 200 μl of PBS and macerated using sterile plastic pestles (Eppendorf). DNA extraction and purification from this sample (hereafter called as hemolymph metagenome) was performed with DNeasy Blood and Tissue Kit (QIAGEN) following manufacturer's instructions.

### DNA sequencing

For gut metagenome DNA Illumina sequencing libraries were prepared using a fragment size of 400 bases and sequenced by Illumina HiSeq2000 platform using a configuration of 200 cycles to obtain pair-end reads of 100 base length. Both library preparation and sequencing were performed at Macrogen Inc. (Korea). The sample yielded a total of 58,146,564 reads. Additionally, DNA from hemolymph metagenome was sequenced using the 454 GS-FLX platform yielding 811,305 single reads.

### Metagenomic fungal ribosomal gene *in silico* reconstruction and characterization

Ribosomal genes from all metagenomic reads were obtained using Parallel-meta 2.4 (Su et al., 2014) algorithm. Eukaryotic ribosomal sequences were recovered using -E option against the SILVA database within an *e*-value of 1 × 10^−10^ cutoff. Fungal 18S rRNA sequences were retrieved from parsing Parallel-meta result tables. Fungal hits were visualized in Krona graphs (Ondov et al., 2011). 18S rRNA gene sequences were recovered from long reads of the hemolymph metagenome (>200 nt), compared to taxonomically related sequences from NCBI using BLASTn 2.2.30+ (Camacho et al., 2009) and used for maximum likelihood phylogenetic analysis. MODELTEST 3.06 was used to select appropriate models of sequence evolution by the AIC model. Model TrN was the best model (A = 0.25409; C = 0.14918; G = 0.20597; T = 0.39076). The ribosomal sequence retrieved was deposited in the GenBank database under the accession number KT351777.

### Gene annotation and purine pathway reconstruction

To eliminate bacterial sequences, all metagenomic reads were mapped to *Wolbachia w*DacA and *w*DacB genomes previously obtained from *D. coccus* metagenome (Ramírez-Puebla et al., 2015) using Bowtie2 2.2.4 (Langmead and Salzberg, 2012). Un-mapped reads were retrieved by Samtools 1.2 (Li et al., 2009). High-quality shotgun unmapped reads longer than 100 nucleotides were used directly for gene prediction and annotation. Gene prediction was performed using FragGeneScan 1.20 (Rho et al., 2010) with –w 0 –p 16 –t illumina_5 (gut, DCoax and DCperu metagenomes) and –t 454_5 (hemolymph-metagenome) parameters. Metabolic annotation was obtained from all putative coding gene predicted using GhostKoala tool from KEGG (Kanehisa et al., 2015). Fungal annotation was obtained by parsing the annotation result table using KEGGREST Bioconductor library (http://bioconductor.org/packages/release/bioc/html/KEGGREST.html). A metabolic pathway of uric acid catabolism was constructed using KEEG Mapper–Search & color Pathway tool (http://www.genome.jp/kegg/tool/map_pathway2.html) from fungal annotation results. All metagenomics reads from gut and hemolymph metagenomes were deposited in GenBank under SRA accession study SRP074499.

Additionally, to extend our metagenomic results we analyzed the two available *Dactylopius* metagenomes from the whole body (here after called DCoax and DCperu metagenome) deposited in GenBank under BioProject PRJNA244295 (Campana et al., 2015). For this, we performed a fungal ribosomal gene *in silico* reconstruction and the annotation of fungal reads related to uric acid catabolism as was described above.

### Phylogenetic analysis

Nucleotide sequences were compared against non-redundant GeneBank library by BLASTn 2.2.30+ (Camacho et al., 2009) and taxonomically related sequences were collected from NCBI. Cultured fungi were identified by ITS and 26S rRNA phylogenies obtained by Maximum likelihood. MODELTEST 3.06 was used to select appropriate models of sequence evolution by the AIC model (Posada, 2008). GTR+I+G (α = 1.772 for gamma distribution; *A* = 0.25778; *C* = 0.23041; *G* = 0.22501; *T* = 0.28681) was the best model for the ITS gene, while GTR + I (α = 0.383 for gamma distribution; *A* = 0.25061; *C* = 0.20735; *G* = 0.29982; *T* = 0.24222) was the best model for 26S rRNA gene. A p-distance among sequences was calculated using DNAdist algorithm from Phylip 3.6 software (Felsenstein, 1989). Limits for genus and species were established at 95 and 97%, respectively. To compare the sequences and quantify the number of fungi operational taxonomical units (OTUs) related with *Dactylopius* spp., a cluster analysis was performed using MOTHUR (Schloss et al., 2009) and ribosomal sequences were clustered at 0.03% distance. All sequences generated from ITS and 26S rRNA of cultured fungi were deposited in the GenBank database under the accessions numbers KM393247 to KM393282 and KT351741 to KT351776, respectively.

### Determination of uric acid and uricase activity in *dactylopius* spp.

Three guts from *D. coccus* and *D. opuntiae* in 1st instar nymph, 2nd instar nymph and adults, as well as eggs from both species, were dissected as mentioned above. Additionally male bodies were resuspended in 200 μl AmplexRed buffer solution. Also, 10 μl of honeydew from *D. coccus* and *D. opuntiae* were resuspended in 100 μl of the same buffer solution. UA and uricase activity were determined using the Amplex® Red Uric Acid/Uricase Assay Kit (Life Technologies Eugene, OR) following the manufacturer's instructions. Means of the UA content as well as uricase activity were compared using two-way ANOVA, and a Tukey-HSD *post-hoc* test was applied for pairwise comparisons between insects. Furthermore, to compare differences in UA content between honeydew and adult female guts a *t*-test was performed. All statistics test were performed using R version 3.1.

### Fungal uricolytic activity

Individual guts and Malpighian tubules, from adults of *D. opuntiae* and *D. coccus* were placed separately in microtubes and macerated with sterile pestles in 200 μl of sterile PBS. Serial 10-fold dilutions from 10^−1^ to 10^−3^ were spread on duplicate plates of MU media (K_2_HPO_4_ 2.5 g l^−1^; KH_2_PO_4_ 5 g l^−1^; MgSO_4•_7 H_2_O 0.2 g l^−1^; MnSO_4_ 0.02 g l^−1^; CaCl_2_ 0.05 g l^−1^; FeSO_4_ 0.05 g l^−1^; uric acid (Sigma) 1.5 g l^−1^; glucose 10 g l^−1^ and agar 15 g l^−1^). Plates were incubated at 28°C in CO_2_ atmosphere generated by BD GasPak EZ Pouch Systems™ for 7 days. Colonies with yeast-like macro and microscopic morphology surrounded with a clear halo (suggestive of uric acid utilization) were counted and colony forming units (CFU) per gut were obtained. All isolates were stored at −70°C. Additionally, uricolytic activity of 37 isolated fungi from *Dactylopius* spp. was tested measuring a degradation halo in YPU (Yeast extract 10 g l^−1^; Peptone 10 g l^−1^, UA 7 g l ^−1^) medium. Enzyme activity was determined as described by Morales-Jiménez et al. (2013). To find out if UA was used by fungi isolates as sole nitrogen source, growth and UA consumption kinetics were performed. Microbial growth was measured quantifying the CFU ml^−1^ for yeast and by weighing the final biomass for molds grown in liquid MU media. UA consumption was quantified by measuring the decrease in absorbance at 295 nm. These results were compared against a standard curve of UA. A Sperman correlation was performed to assess a negative correlation and differences in UA consumption in relation to time.

### Antifungal treatment

A group of 15 first instar nymphs of *D. opuntiae* was fed on a prickly pear pad of *Opuntia ficus-indica* injected with 5 ml of 20 μg ml^−1^ antifungal cocktail of Ketoconazol (Sigma), Anfotericine B (Sigma), and Fludioxonil (Sigma). Fleshy leaves were injected weekly for 4 weeks and then female insects were removed. *O. ficus-indica* leaves without antifungal were similarly infested and used as negative controls. After treatment, a pool of six individuals of each leaf was used to measure differences in dry weight, UA content and uricase activity. Five replicates of this experiment were performed. UA content, uricase activity and dry weight data were compared between controls and treatments using a *t*-test.

### Fluorescent *in situ* hybridization (FISH)

FISH was performed as previously described by Koga et al. (2009) with slight modifications. Ninety-day old *D. coccus* and *D. opuntiae* were collected. Malpighian tubules, as well as ovaries and embryos (25 from *D. coccus* and 20 from *D. opuntiae*) were dissected as described above. These organs were embedded in 3% agarose and treated as described by Rosas-Pérez et al. (2014). The oligonucleotide probe used was Cy5-Cry851 (5′-TGATGCGAGT TTCTGCTATC-3′), which targets 26S rRNA of *Cryptococcus saitoi* (designed for this work). After washing with PBS the samples were stained with 2.4 μg ml^−1^ of DAPI and mounted with citifluor antifade solution. To confirm probe specificity, control experiments were performed with no probe and RNAse digestion. The samples were observed under an Olympus FV100 Multi-photonic confocal microscopy. Images were processed using FIJI 2.0.0 software (Schindelin et al., 2012).

## Results

### Culture-dependent and culture-independent analyses of fungal communities

**Figure 1:**
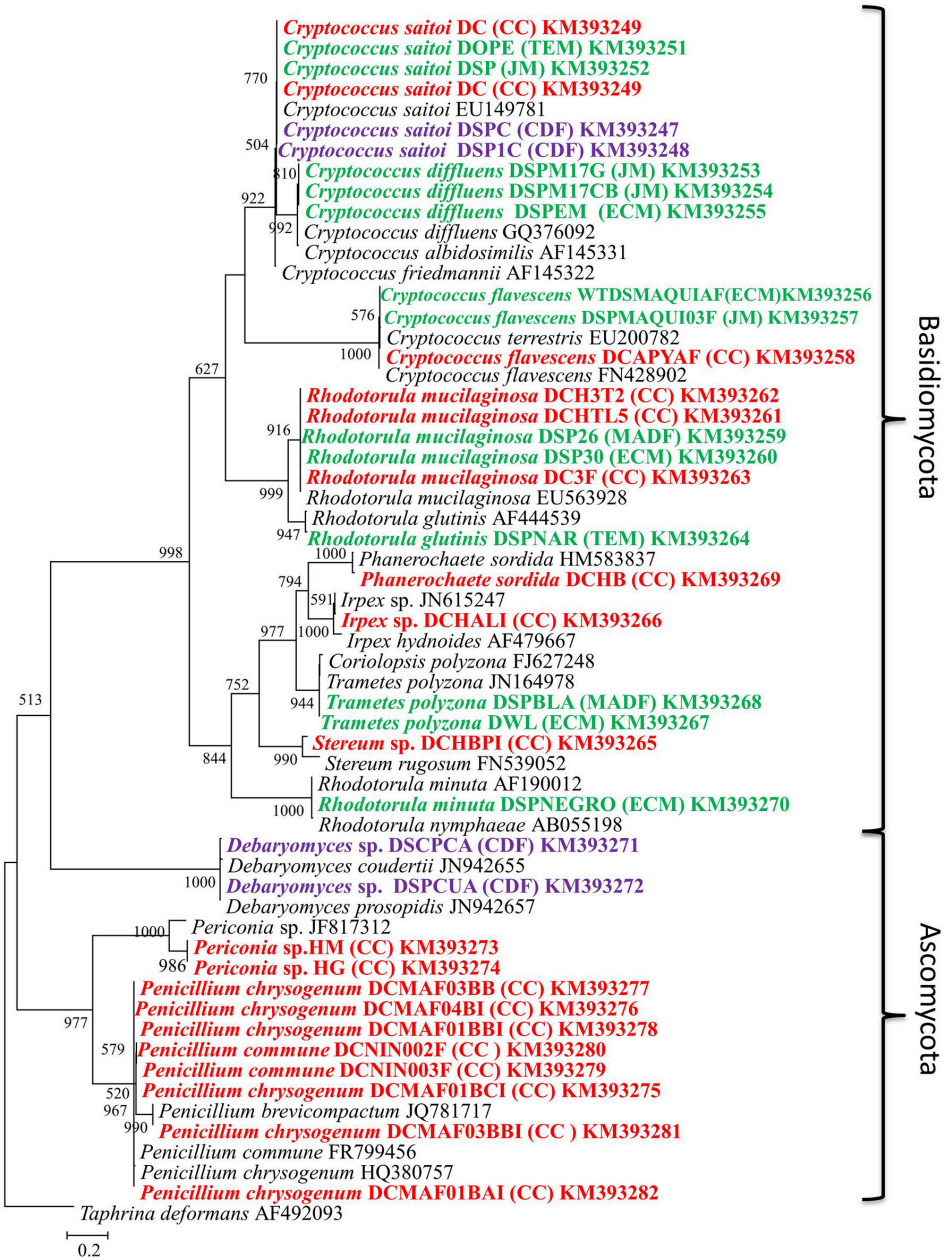
**Maximum likelihood tree (−ln L = −5579.17063) of fungi isolated from different species of *Dactylopius* spp**. The ITS sequence of *Taphrina deformans* was used as outgroup. Scale bar indicates 2% estimated sequence divergence. Bootstrap support values ≥ 50% are indicated. Colors mean different *Dactylopius* species. Red, *D. coccus*; green, *D. opuntiae;* and purple, *D. confusus*. Letters in parentheses show the collect site (Table 1).

A total of 37 fungal isolates were cultured from *D. coccus, D. opuntiae*, and *D. confusus*. Isolates were obtained from guts, whole bodies and ovary samples (Table 2). Nucleotide sequences of 26S rRNA genes and ITS regions from different morphotypes corresponded to 14 OTUs. 26S rRNA and ITS phylogenetic analyses showed sequences belonging to Ascomycota and Basidiomycota with *Rhodotorula, Cryptococcus* and *Penicillium* as the most frequent genera (Figure 1; Supplementary Figure 1). Fungal species like *Rhodotorula mucilaginosa* and *Cryptococcus saitoi* were present in the three *Dactylopius* species sampled, whereas *Trametes polizona* was present in *D. coccus* and *D. opuntiae* (Table 2). Three filamentous fungi had an ITS sequence identity of 77.6 and 88.9% to *Stereum* sp. and *Periconia* sp. (DCHG and DCHM) respectively (Figure 1; Table 2). In 26S rRNA phylogenies, the closest related sequences of these novel fungi were *Phlebiopsis flavidoalba* (DCHBPI) with 97.8% identity and *Periconia macrospinosa* (DCHG and DCHM) with 93.74% identity (Table 2, Supplementary Figure 1). Likewise, from *D. coccus* we could isolate the mold *Penicillium* from 1st instar nymphs (*n* = 3) and males (*n* = 5) but not from adult females (Figure 1; Table 2; Supplementary Figure 1).

**Table 2:** **Fungi associated with different *Dactylopius* species in culture-dependent analysis**.

**Insect host**	**Isolate name**	**Most related fungi ITS sequence from GenBank (identity %)**	**Most related fungi 26S rRNA sequence from GenBank (identity %)**	**OTU Number**	**Morphology Yeast (Y) Mold (M)**	**Isolated from: Ovary-eggs (O) Gut (G) Whole body (W)**	**Insect host stage**
*Dactylopius coccus*	DCHTL5	*Rhodotorula mucilaginosa* EU56392 (100)	*Rhodotorula mucilaginosa* DQ832198 (100)	1	Y	G	Adult female
	DC3F				Y	O	Egg
	DCH3T2				Y	W	Adult female
	DC	*Cryptococcus saitoi* EU149781 (100)	*Cryptococcus saitoi* JX188127 (100)	4	Y	W	Adult female
	DCAPYAF	*Cryptococcus flavescens* FN428902 (99.76)	*Cryptococcus flavescens* FJ743610 (98.5)	5	Y	W	Adult female
	DCHBPI	*Stereum* sp. GQ999353 (77.58)	*Phlebiopsis flavidoalba* EU118662 (97.8)	9	M	W	Adult female
	DCALI	*Irpex* sp. JN615247 (99.78)	*Irpex lacteus* JN710547 (99.8)	8	M	G	Adult female
	DCHBP	*Trametes polyzona* JN164978 (99.77)	*Trametes polyzona* JN164790 (100)	7	M	G	Adult female
	HG	*Periconia* sp. JN164978 (88.85)	*Periconia macrospinosa* JN859484 (93.74)	11	M	O	Egg
	HM				M	O	Egg
	DCHB	*Phanerochaete sordida* HM583837 (98.60)	*Phanerochaete sordida* HM595608 (97.8)	10	M	G	Adult female
	DCNin003F	*Penicillium commune* FR799456 (99.06)	*Penicillium nalgiovense* JQ434685 (100)	13	M	W	1st instar
	DCNin002F				M	G	1st instar
	DCNIN01F	*Penicillium chrysogenum* HQ380757 (99.76)	*Penicillium cavernicola* JQ434692 (100)	14	M	W	1st instar
	DCMAF01BCI				M	W	Adult male
	DCMAF04BI				M	W	Adult male
	DCMAF01BAI				M	W	Adult male
	DCMAF01BBI				M	W	Adult male
	DCMAF03BB				M	W	Adult male
*Dactylopius confusus*	DSPC	*Cryptococcus saitoi* EU149781 (100)	*Cryptococcus saitoi* JX188127 (100)	4	Y	W	Adult female
	DSCP1C				Y	G	Adult female
	DSP26	*Rhodotorula mucilaginosa* EU56392 (100)	*Rhodotorula mucilaginosa* DQ832198 (100)	1	Y	G	2nd instar
	DSPCUA	*Debaryomyces prosopidis* JN942657 (100)	*Debaryomyces hansenii* AB470569 (100)	12	Y	G	Adult female
	DSPA				Y	G	Adult female
*Dactylopius opuntiae*	DSPNAR	*Rhodotorula glutinis* AF444539 (100)	*Rhodotorula glutinis* KC494740 (100)	2	Y	G	Adult female
	DSP30	*Rhodotorula mucilaginosa* EU56392 (100)	*Rhodotorula mucilaginosa* DQ832198 (100)	1	Y	G	2nd instar
	DSPNEGRO	*Rhodotorula minuta* AF190012 (100)	*Rhodotorula minuta* EU583491 (99.8)	3	Y	G	Adult female
	DWL	*Trametes polyzona* JN164978 (99.77)	*Trametes polyzona* JN164790 (100)	7	Y	W	Adult female
	DSPMGT17CB	*Cryptococcus diffluens* GQ376092 (99.58)	*Cryptococcus diffluens* AF335981 (100)	6	Y	G	Adult female
	DSPEM				Y	G	2nd instar
	DSPM17G				Y	G	Adult female
	DOP	*Cryptococcus saitoi* EU149781 (100)	*Cryptococcus saitoi* JX188127 (100)	4	Y	W	Adult female
	DOPE				Y	O	Egg
	DSP				Y	W	1st instar
	WTDSMAQUIAF	*Cryptococcus flavescens* FN428902 (100)	*Cryptococcus flavescens* FJ743610 (98.5)	5	Y	G	Adult female
	DSPMAQUI03F				Y	G	Adult female
	DSPBLA	*Trametes polyzona* JN164978 (99.77)	*Trametes polyzona* JN164790 (100)	7	M	G	Adult female

**Figure 2:**
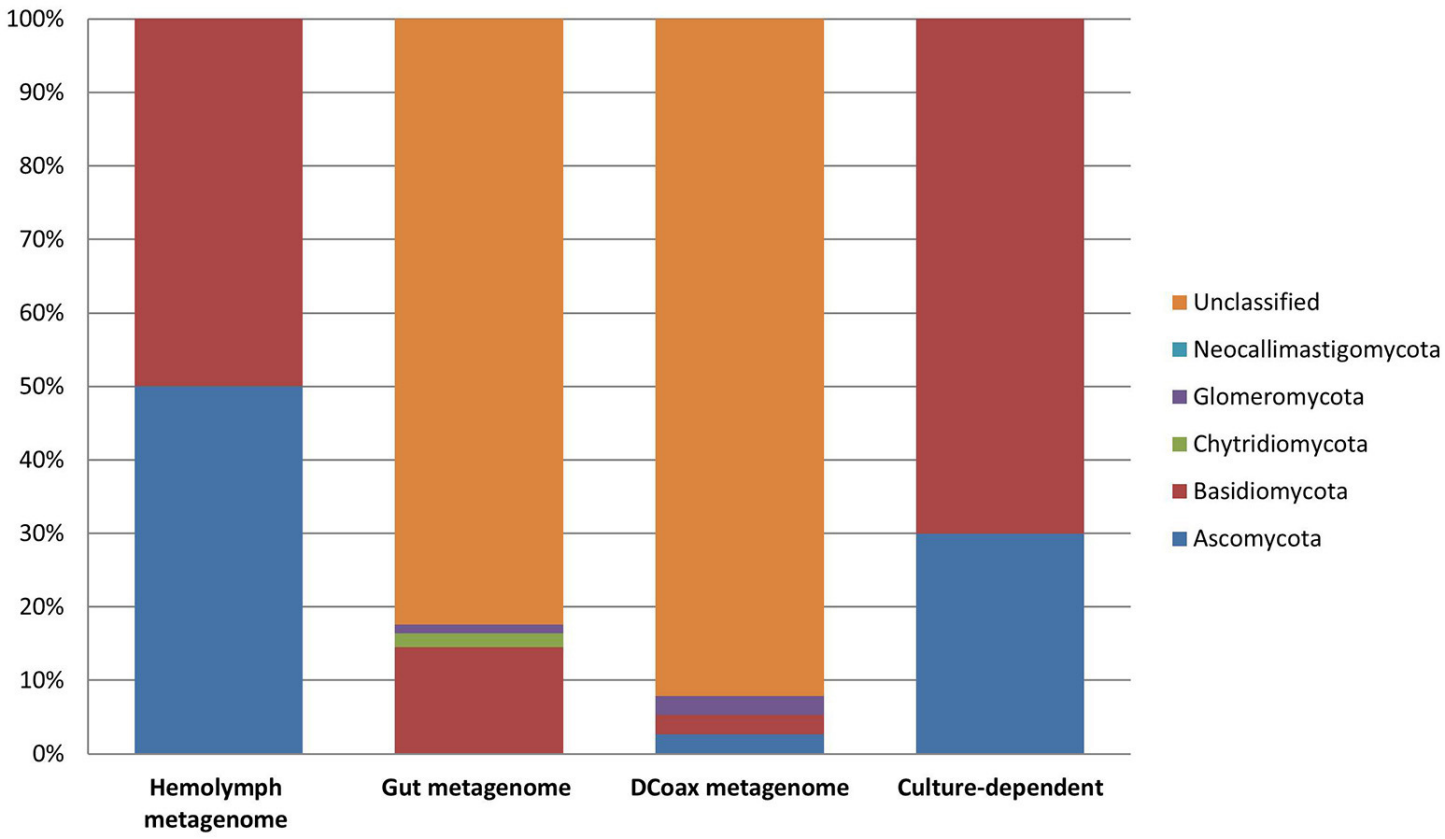
**Fungal composition assessed by taxonomic classification of ribosomal rRNA markers from metagenomic (culture-independent) and culture-dependent analyses of *D. coccus***.

From the metagenomic data of the hemolymph and gut metagenomes, fungal 18S rRNA gene sequences were detected. Hemolymph metagenome sequences were assigned particularly to *Sebacina vermifera, Bullera ninhbinhensis* (Basidiomycetes), and *Candida lignicola* (Ascomycetes; Figure 2; Supplementary Data Sheet 1). In congruence, a phylogenetic reconstruction of 18S rRNA (~200 nt) from this sample showed the presence of *Pichia anomala* (100% identity) in *Dactylopius* hemolymph (Supplementary Figure 2). In gut metagenome, we found sequences related to Basidiomycota, particularly to the Sebacinaceae family (*Craterocolla* sp. and *Sebacina* sp.) and Ustilaginaceae family (*Rhodosporidium* sp.), as well as sequences related to Chytridiomycota and Glomeromycota phyla (Figure 2; Supplementary Data Sheet 1). Remarkably, most of the fungal sequences obtained by the metagenomic analysis were associated with uncultured and unclassified fungi (Figure 2; Supplementary Data Sheet 1). Analysis of DCoax metagenome showed sequences related to Basidiomycota (*Agaricus bisporus* and *Thanatephorus cucumeris*), Ascomycota (*Blastobostrys adeninivorans* and *Candida* sp.), Glomeromycota and some unclassified fungi (Figure 2; Supplementary Data Sheet 1). From DCperu metagenome the only fungal species detected was *Candida* sp.

### Metagenomic annotation of fungal genes involved in uric acid catabolism

**Figure 3:**
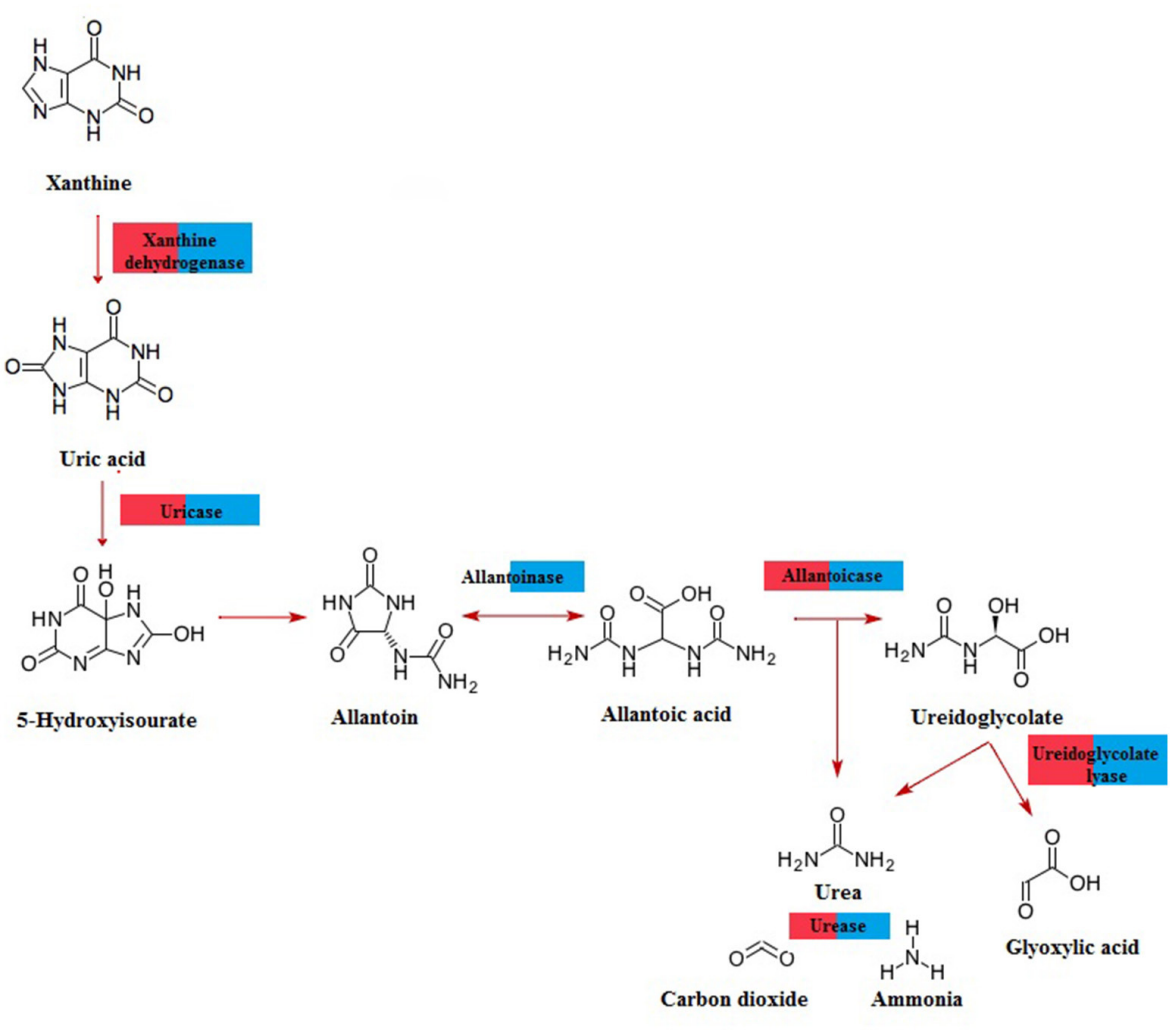
**Pathway for purine catabolism of fungal genes predicted from *D. coccus* gut (blue) and hemolymph (red) metagenomes**.

A total of 518,258 open reading frames (ORFs) were predicted from the hemolymph metagenome and 20,136,058 ORFs from the gut metagenome. From those, only 2,874 and 66,502 corresponded to fungal ORFs, respectively. Metabolic annotation of these fungal ORFs revealed genes related to UA metabolism (Figure 3). Particularly, we detected the presence of 20 and 85 fungal genes involved in UA catabolism from hemolymph and gut metagenome, respectively (Supplementary Table 1). All coding genes for xanthine degradation to urea were present in gut metagenome whereas in hemolymph metagenome we did not find any allantoinase fungal genes (Figure 3). From DCoax metagenome a total of 8,911,722 ORFs were estimated and 8,901,672 were properly annotated by Ghost-KOALA, from which 262,623 corresponded to fungal sequences. We found 128 putative genes involved in uric acid catabolism in this metagenome (Supplementary Table 2). From the DCperu metagenome, 8,619,769 ORFs were predicted; 8,611,041 had a functional annotation and 226,810 belonged to fungal sequences. A total of 101 putative genes of uric acid catabolism were present in this sample (Supplementary Table 3). As in gut metagenome, all genes for xanthine catabolism to urea were found in DCoax and DCperu metagenomes (Supplementary Figure 3).

### UA and uricase activity in *Dactylopius* spp. guts

**Figure 4:**
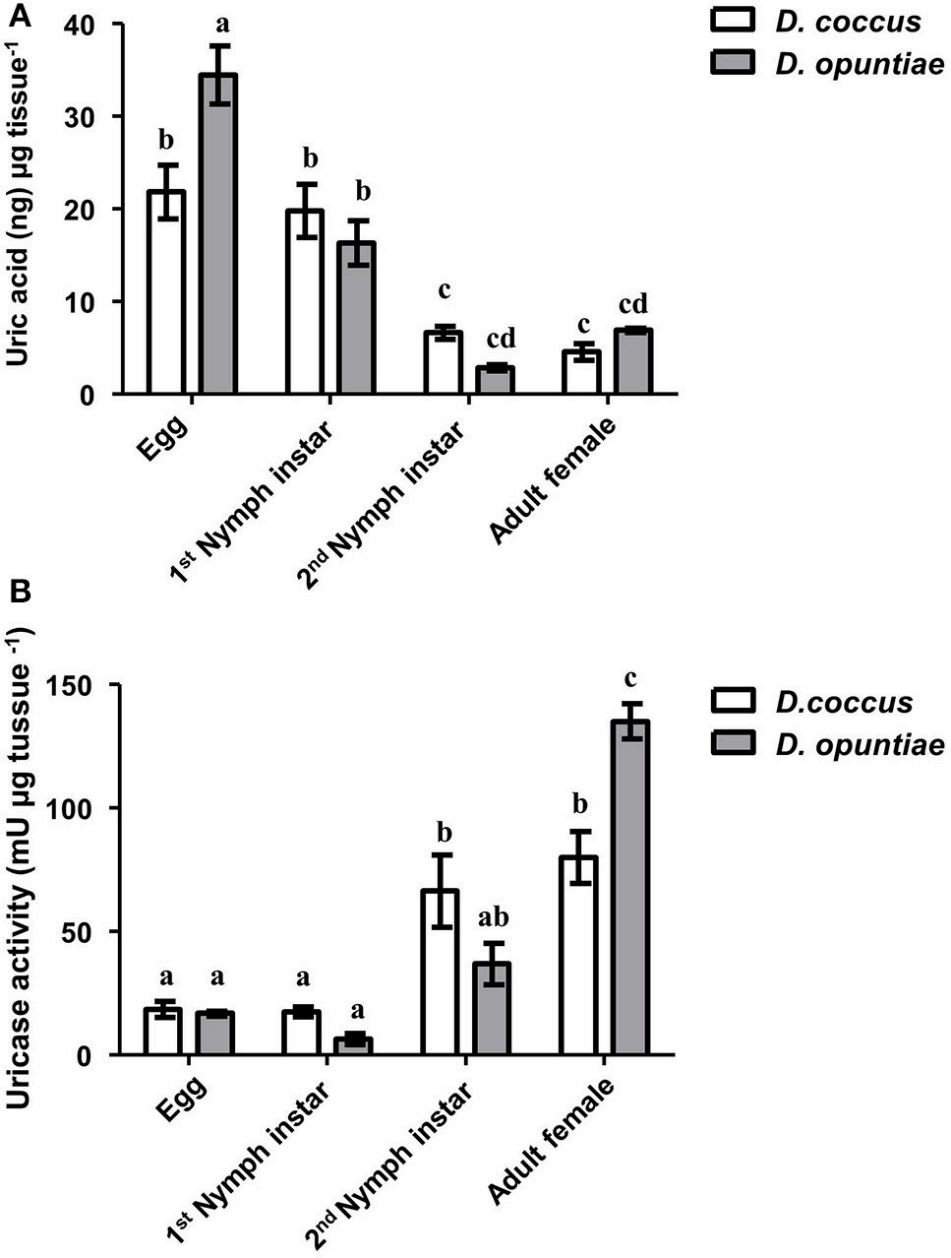
**Uric acid and uricase activity during different life stages of *D. coccus* and *D. opuntiae*. (A)** Uric acid content in ng per microgram tissue (Two way-ANOVA *P* = 0.1978; *F* = 1.73 d.f. 1). **(B)** Uricase activity in milli-Units per microgram tissue (Two way-ANOVA *P* = 0.5585 *F* = 0.3496 d.f. 1). Values are shown as means ± SE of five independent experiments. Mean values with different letters are significantly different (Tuckey-HDS test *P* < 0.01).

UA and uricase activities were detected in *D. opuntiae* and *D. coccus* extracts where the changes in UA concentration depended on the insect developmental stage (Figure 4A). The highest amount of UA was present in eggs of both species (21.87 ± 2.91 and 34.49 ± 3.11 ng μg^−1^ tissue, respectively; Supplementary Table 4) whereas the lowest was in *D. coccus* adult male, *D. coccus* female and in *D. opuntiae* 2nd instar nymph (4.49 ± 0.38; 4.61 ± 0.91 and 2.91 ± 0.32 ng μg^−1^ tissue respectively; Supplementary Table 4).

*Post-hoc* comparison using Tukey-HSD test showed significant differences in UA content among eggs, 1st instar nymph, and adults in both species, although no significant difference was seen between 2nd nymph instar and adult (Figure 4A).

Urate oxidase or uricase (EC 1.7.3.3 or UOX) is a homo-tetramer that catalyzes the conversion of UA and molecular oxygen to 5-hydroxyurate and hydrogen peroxide (Gabison et al., 2008). In our results, this enzyme showed high activity in adult females of both *Dactylopius* species (80 mU μg^−1^ tissue for *D. coccus* and 135 mU μg^−1^ tissue for *D. opuntiae*; Figure 4B; Supplementary Table 4). *Post-hoc* test showed significant differences in uricolytic activity in all stages (Figure 4B). The content of uric acid in adult's honeydew in both scale species was low, 0.18 ± 0.05 and 0.58 ± 0.05 ng μl^−1^ in *D. coccus* and *D. opuntiae*, respectively. A *t*-test showed a significant difference between UA content in honey dew and adults gut (*D. coccus P* = 0.0006; *t* = 4.856; *df* = 8; *D. opuntiae P* < 0.0001; *t* = 26.85; *df* = 8), moreover no urate oxidase activity was detected in these samples. This supports the idea that UA is metabolized inside the insect.

### Uricolytic fungi associated with *Dactylopius*

**Figure 5:**
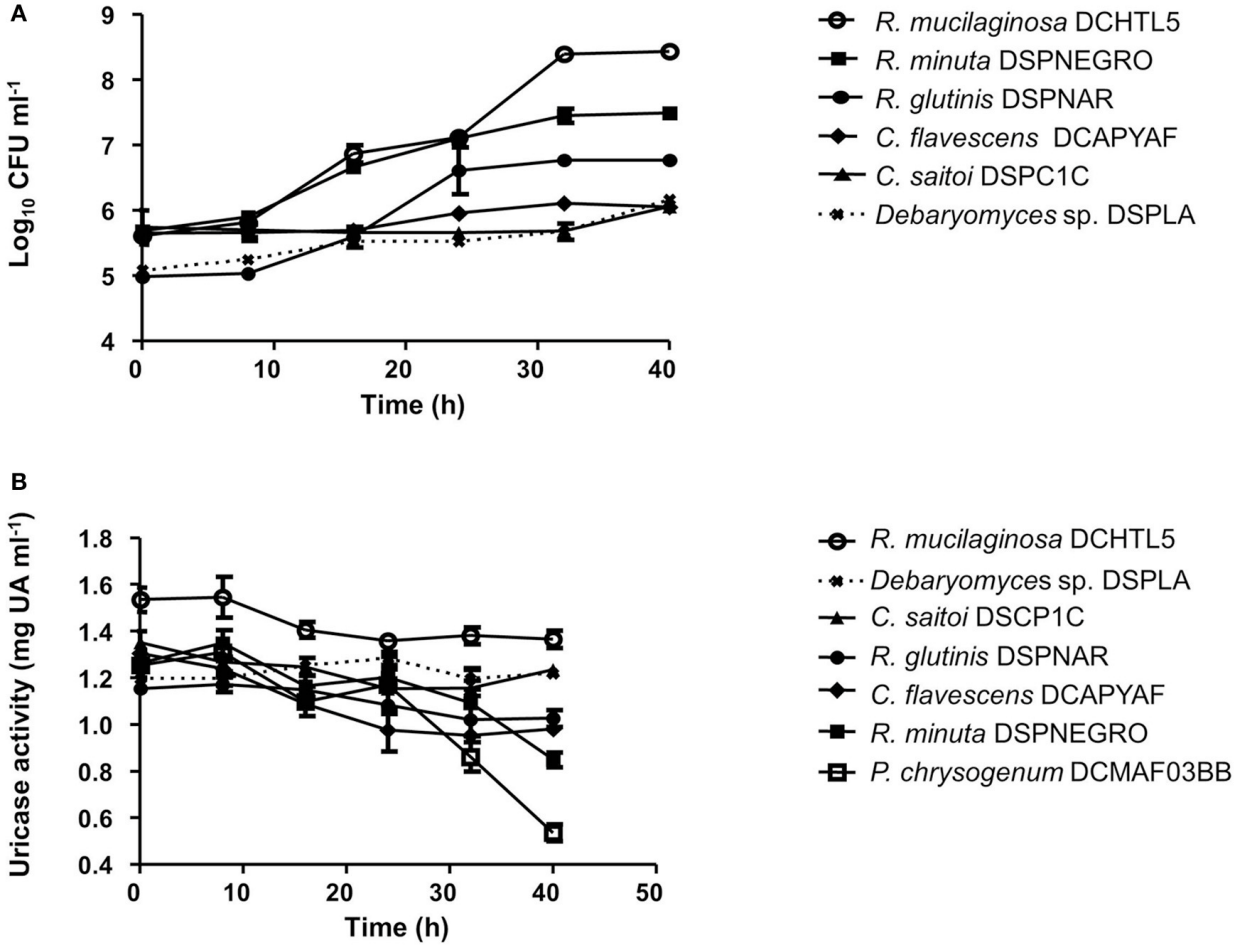
**(A)** Growth kinetics of uricolytic yeast associated with *Dactylopius* spp. using uric acid as sole nitrogen source. **(B)** Uric acid consumption kinetics of uricolytic yeast associated with *Dactylopius* spp. using uric acid as sole nitrogen source. Values are shown as means ± SEM of three independent experiments.

The number of uricolytic yeast CFUs in MU from *D. opuntiae* gut was estimated in 4.1 × 10^2^ ± 0.74 × 10^2^ CFU gut^−1^. The isolates *C. flavescens* DCPYAF01, *R. mucilaginosa* DCHTL5, *R. minuta* DSPNEGRO, *R. glutinis* DSPNAR, *C. saitoi* DSPCUB, and the mold *Penicillium* sp. DCFM03BB (Figure 1; Table 2), were capable of growth and consumption of UA as sole nitrogen source (Figures 5A,B; Table 3). The maximum consumption rates were with *Penicillium* sp. DCMAF03BB and *R. minuta* DSPNEGRO (717.9 ± 27.05 and 414.8 ± 66.43 μg of UA respectively; Table 3). *Debaryomyces* sp. DSPA showed no significant growth and there was no evidence for UA uptake by this strain (Figures 5A,B; Table 3).

**Table 3:** **Uric acid consumed as sole nitrogen source by fungi isolated from *Dactylopius***.

**Isolate**	**Uric acid consumed (μg ml^−1^)**	**Sperman correlation *R*-value**	***P*-value**
*Rhodotorula glutinis* DSPNAR	127.6 ± 42.54	–0.922	0.0045
*Cryptococcus saitoi* DSPC1C	119.9 ± 62.16	–0.725	0.0515
*Rhodotorula minuta* DSPNEGRO	414.8 ± 66.43	–0.897	0.0128
*Rhodotorula mucilaginosa* DCHTL5	170.5 ± 89.54	–0.867	0.0127
*Cryptococcus flavescens* DCAPYAF	323.5 ± 37.34	–0.925	0.0041
*Debaryomyces* sp. DSPA	0.0 ± 0.0	0.221	0.3372
*Penicillium* sp. DCM03BB	717.9 ± 27.05	–0.892	0.0085

### Antifungal effects on UA concentration and uricase activity in *D. opuntiae*

**Figure 6:**
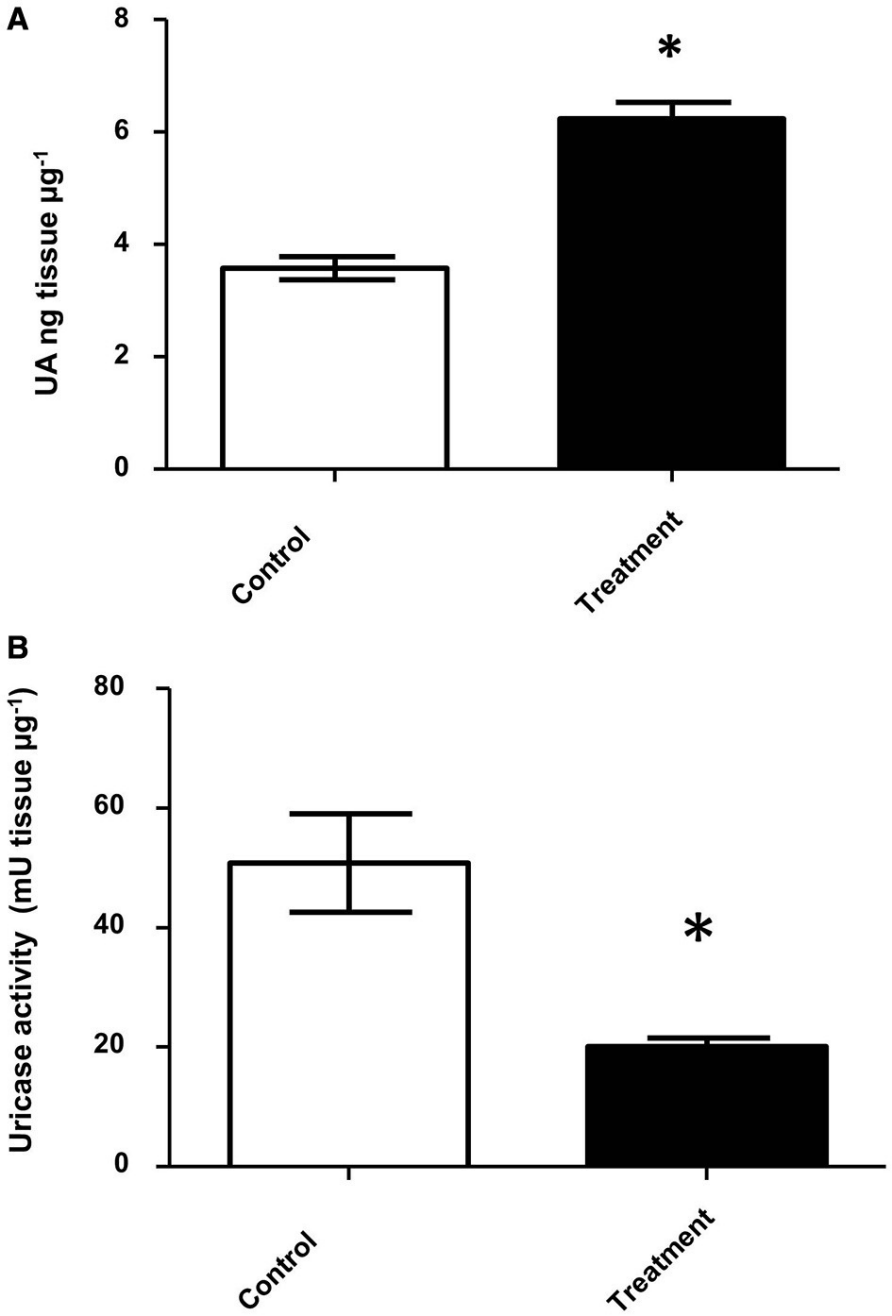
**Uric acid and uricolytic activity in gut of insects treated with antifungals. (A)** Uric acid content in *D. opuntiae* gut. ^*^ shows significant difference between treatments (*t*-test *t* = 10.05; *df* = 2; *P* = 0.0098). **(B)** Uricolytic activity in *D. opuntiae* gut. ^*^ shows significant difference between treatments (*t*-test *t* = 3.671 *df* = 4, *P* = 0.0214). Values are shown as means ± SE of five independent experiments.

After four weeks with antifungal treatment *D. opuntiae* weight was significantly lower in comparison to the controls (2.50 ± 0.15 and 0.58 ± 0.12 mg respectively; *t* = 6.954; *df* = 4; *P* = 0.0201; Supplementary Figure 4). Uric acid concentration was significantly higher in fungicide treated insects vs. controls (6.25 ± 0.28 and 3.58 ± 0.21 UA ng μg^−1^ tissue ^1^ respectively; Figure 6A). Additionally, uricase activity was significant lower in antifungal treatments than in controls (20.20 ± 1.35 and 50.91 ± 8.26 mU tissue μg^−1^, respectively; Figure 6B).

### *Cryptococcus saitoi* localization in *Dactylopius*

**Figure 7:**
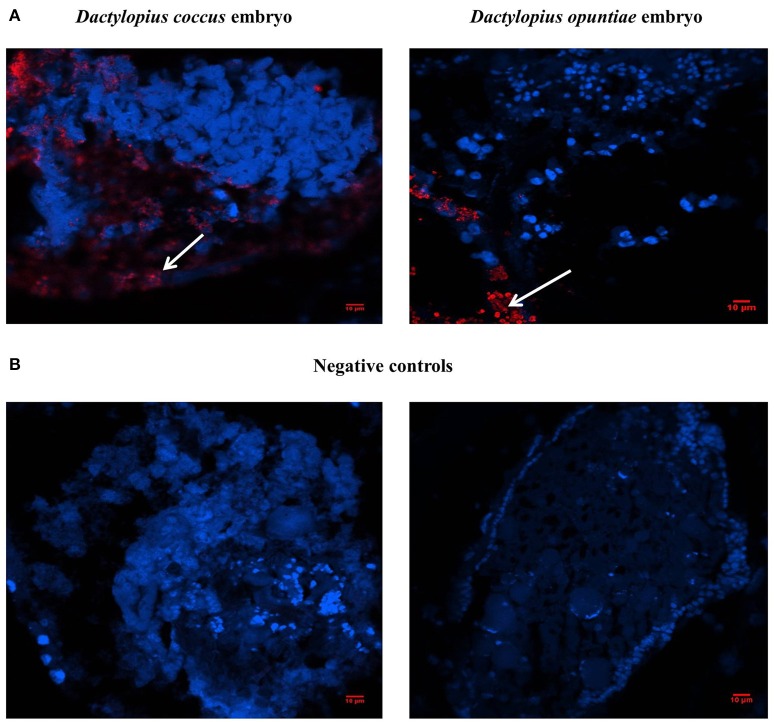
**Localization of *Cryptococcus saitoi* inside *Dactylopius***. In the fluorescence *in situ* hybridization (FISH) images, blue and red signals indicate insect nuclear DNA and fungi respectively. **(A)** Left *D. coccus* embryo; right *D. opuntiae* embryo. **(B)** No-probe controls; Left *D. coccus* embryo; right *D. opuntiae* embryo. White arrows show *C. saitoi* signal.

Fluorescent *in situ* hybridization of *D. coccus* and *D. opuntiae* showed the presence of *C. saitoi* in embryos of both species (Figures 7A,B). Of 25 embryos of *D. coccus* and 20 of *D. opuntiae*, 17 (68%) and 14 (70%) contained the fluorescent signal. FISH analysis showed that *C. saitoi* fungi were on the egg surface. Additionally, *C. saitoi* was observed by FISH in a distal part of the Malpighian tubules in *D. coccus* (Supplementary Figure 5).

## Discussion

A comprehensive study of the fungal community associated with *Dactylopius* is presented here, where different species in four fungal phyla were found by culture and culture-independent analyses. *C. saitoi* and *R. mucilaginosa* were found in most female samples (Figure 2) while *Penicillum* was the only fungus found in males (Figure 1; Table 2; Supplementary Figure 1). *Penicillium* has been associated with other insects such as bees, beetles, termites, and as well as in *Triatoma* sp. guts (Batra et al., 1973; Lage-Moraes et al., 2001; Pérez et al., 2003). The cultured fungi obtained belonging to *Rhodotorula, Cryptococcus, Trametes, Penicillium*, and *Debaryomyces* (Figure 1; Supplementary Figure 1) were previously found in other phytophagous insects (Jones et al., 1999; Guevara et al., 2000; Suh et al., 2001; Ganter, 2006; Kobayashi et al., 2007). Particularly in the scale insect *Saissetia oleae, Cryptococcus*, and *Rhodotorula* yeasts were isolated from the gut and reproductive organs (Zacchi and Vaughan-Martini, 2003). Similarly, in the reproductive tissues and guts from *D. coccus* and *D. opuntiae*, we found *Cryptococcus* and *Rhodotorula* by a culture dependent approach and by FISH (Figure 7; Table 2; Supplementary Figure 5). In culture we also found *P. flavidoalba* (DCHBPI), *Periconia macrospinosa* (DCHG and DCHM) and *Irpex lacteus* (DCALI) which, to our knowledge, have not been previously isolated from insect's inner tissues. In this work ITS and 26S rDNA markers were used for culture-fungi identification and in few cases genus or species assignment differed depending on the marker used (Table 2), indicating that single gene phylogenetic stories are not fully reliable and a better sample of the genome is needed in novel groups.

Most of the fungal ribosomal sequences from the female metagenomic analyses belonged to uncultured or non-classified fungi. With ~100–300 base pair reads an accurate classification may be difficult. Additionally, fungal sequences are underrepresented in metagenomics because of limited information in databases used for the analysis and problems in fungal DNA extraction from different samples (Lindahl and Kuske, 2013; Escobar-Zepeda et al., 2015). However, members of Chytridiomycota and Glomeromycota phyla (Figure 2; Supplementary Data Sheet 1) were recovered form *D. coccus* metagenomes. There are reports of entomopathogenic Chytridiomycota associated with elm bark beetles, blackflies, and aquatic dipteran larvae (Humber et al., 1990; Powell, 1993), but not in scale insects. Glomeromycota is a phylum of asexual fungi from arbuscular mycorrhiza of plants, they are obligate endosymbionts and cannot be grown in pure culture in the absence of their plant host (Hempel et al., 2007; Gianinazzi-Pearson and Van Tuinen, 2012). Interestingly, there are no reports of this fungal phylum associated with insects, although some sequences related to mycorrhizal fungi have been found in other habitats like the human oral cavity (Ghannoum et al., 2010; Cui et al., 2013). In *Dactylopius* we found sequences of Glomeromycota in gut and whole body (Supplementary Data Sheet 1). It is tempting to speculate that its presence could mediate a close interaction between insects and their host plant. This is the first report of Glomeromycota in insects.

Sequences of *Candida*, which we did not recover in cultures (Figure 1; Table 2; Supplementary Figure 1), were found in all female *Dactylopius* metagenomes (Supplementary Data Sheet 1; Supplementary Figure 2). Species of *Candida* have been isolated from insect guts as well as in mycetocytes of other hemipterans (Gibson and Hunter, 2005; Vega and Blackwell, 2005; Suh et al., 2008; Hughes et al., 2011).

Additionally, we report here the presence of uricolityc fungi associated with *Dactylopius* spp. Nitrogen content in *O. ficus-indica* cladodes is around 0.5–1% of wet weight (Stintzing and Carle, 2005). Meanwhile in *Dactylopius* this element constitutes about 32% of wet weight (Gómez-Hernández, 2006). This means that *Dactylopius* has to accumulate 30 times the nitrogen present in the cactus. It is known that N_2_ recycling by UA catabolism provides nitrogen to plant feeding insects (Potrikus and Breznak, 1981; Sasaki et al., 1996; Morales-Jiménez et al., 2013; Patiño-Navarrete et al., 2014). However, bacteria are often mentioned as major recyclers in these scenarios and only in the brown plant hopper (*Nilaparvata luggens*) it has been shown that many unicellular fungi symbionts called yeast-like symbionts (YLS) are involved in insect UA metabolism (Sasaki et al., 1996). Plant hoppers produce and store UA when fed nitrogen-rich diets, but when nitrogen is limited their YLS mobilize the stored UA using the enzyme uricase (EC:1.7.3.3). This process may turn UA into amino acids for insects. Yeast isolates from *D. coccus* and *D. opuntiae* females as well as the mold *Penicillium* from *D. coccus* males were capable of metabolizing UA as sole nitrogen source (Figures 5A,B; Supplementary Table 4) There are reports for UA catabolism in *Cryptococcus* and *Penicillium* (Allam and Elzainy, 1969; Lee et al., 2013) but to our knowledge there are no reports for uricolytic *Rhodotorula* (Middelhoven et al., 1985). In termites (*Reticulotermes flavipes*) and in bark beetles (*Dendroctonus valens* and *Dendroctonus rhizophagus*) uricolytic microorganisms have been isolated from their guts (Potrikus and Breznak, 1980; Morales-Jiménez et al., 2013), in agreement most of the *Dactylopius* uricolytic fungi come from the alimentary canal (Figures 5A,B; Table 2). FISH analysis showed the presence of *Cryptoccocus* (uricolytic yeast) in Malpighian tubules of *D. coccus* (Supplementary Figure 5). Additionally, metagenomic analysis of guts and hemolymph of *D. coccus* and whole body of other *D. coccus* revealed the presence of fungal genes involved in UA catabolism (Figure 3; Supplementary Figure 3; Supplementary Tables 2, 3). Uricase catalyzes the first step in UA catabolism (Gabison et al., 2008). Even though putative genes for uricase were present in all metagenomes analyzed, there was only one ORF codifying for this enzyme in hemolymph metagenome; meanwhile in the gut metagenome 18 of these genes were found (Supplementary Table 2). This supports the idea that UA could be metabolized by fungi in *Dactylopius* gut, as in other insects, rather than directly in hemolymph. Besides, putative fungal genes for allantoinase, allantoicases, and ureases were also found. This suggests that UA can be catabolized to urea and ammonia by fungi (Figure 3; Supplementary Figure 3). It is known that in silkworm *Bombix mori* and in the larvae of the bruchid beetle *Caryedes brasiliensis* urea can be incorporated into insect proteins as an alternative nitrogen source (Hirayama et al., 1999). In *Dactylopius* uric acid could be metabolized into urea by their associated fungi and then used as nitrogen by its insect host.

Different levels of UA during life stages have been detected in other Hemiptera. Particularly in *Parastrachia japonensis*, UA is higher before copulation and during ovarian development and lower in nymph stages (Kashima et al., 2006). In contrast, in *Dactylopius* we found that UA is higher in nymphs as compared to adults (Figure 4A; Supplementary Table 4). Uricase activity was detected in *Dactylopius* guts in all life stages, in contrast this enzyme is absent in the majority of insects (Pant, 1988). However, some insect symbionts present uricase activity (Potrikus and Breznak, 1981; Hongoh and Ishikawa, 2000). In the shield bug *P. japonensis* treatment with antibiotics produce a reduction in uricolytic activity and in amino acid concentration in hemolymph (Kashima et al., 2006). In *Dactylopius*, antifungal treatment showed a similar significant decrease of uricase activity (Figure 6B), additionally UA concentration was higher in those insects treated (Figure 6A). As mentioned, the metagenomic approach revealed fungal uricase genes (Figure 3; Supplementary Figure 3; Supplementary Tables 1–3), that in addition to the experimental evidence of UA accumulation and lower uricolytic activity in antifungal treated insects (Figures 6A,B), suggest that the uricase detected in the enzymatic assay on *Dactylopius* (Figure 4B; Table 3) may come from their associated fungi. In conclusion fungi associated to *Dactylopius* could recycle nitrogen in order to supply deficiencies in their diet.

## Author contributions

The experiments were conceived and designed by AV, AS, MR, and EM, and were conducted and analyzed by AV and AS. All authors contributed to interpreting the results and writing the article.

## Funding

This work was supported by Consejo Nacional de Ciencia y Tecnologia grant 154453 and graduate student (AVPL) grant 331625.

### Conflict of interest statement

The authors declare that the research was conducted in the absence of any commercial or financial relationships that could be construed as a potential conflict of interest.
