# Supplementary material for: Fungal Community Associated with Dactylopius (Hemiptera: Coccoidea: Dactylopiidae) and Its Role in Uric Acid Metabolism
Source: Front Microbiol. 2016 Jun 23;7:954. doi: 10.3389/fmicb.2016.00954 (PMC4917543; doi:10.3389/fmicb.2016.00954)
Supplement: Supplementary file 4 [file Table4.DOCX]

Supplementary Table 4. Uric acid content and uricolytic activity in guts of *D. coccus* and *D. opuntiae* in different life stages.

| Insect species | Life stage | Uric acid ng µg^-1^ tissue | Uricolytic activity mU µg^1^ tissue |
| --- | --- | --- | --- |
| *D. coccus* | Egg | 21.87 ± 2.91 | 18.58 ± 3.237 |
|  | 1^st^ instar nymph | 19.86 ± 2.90 | 17.66 ± 2.005 |
|  | 2^nd^ instar nymph | 6.69 ± 0.70 | 66.59 ± 14.63 |
|  | Adult female | 4.61 ± 0.91 | 80.11 ± 10.43 |
|  | Adult male | 4.49 ± 0.38 | 13.19 ± 0.73 |
|  | Honeydew | 0.18 ± 0.05 | 0.00 ± 0.00 |
| *D. opuntiae* | Egg | 34.49 ± 3.11 | 16.88 ± 1.05 |
|  | 1^st^ instar nymph | 3.11 ± 2.40 | 6.55 ± 2.30 |
|  | 2^nd^ instar nymph | 2.91 ± 0.32 | 37.05 ± 8.33 |
|  | Adult female | 6.94 ± 0.23 | 135.20 ± 7.13 |
|  | Honeydew | 0.58 ± 0.05 | 0.00 ± 0.00 |
