# Supplementary material for: Fungal Community Associated with Dactylopius (Hemiptera: Coccoidea: Dactylopiidae) and Its Role in Uric Acid Metabolism
Source: Front Microbiol. 2016 Jun 23;7:954. doi: 10.3389/fmicb.2016.00954 (PMC4917543; doi:10.3389/fmicb.2016.00954)
Supplement: Supplementary file 5 [file DataSheet1.ZIP › Supplementary_DataSheet1/Supplementary_DataSheet1.html]

Javascript must be enabled to view this page.

members
magnitude
magnitudeUnassigned

Hemolymph
Gut
DCoax\_metagenome

316776

3167

3167

1

1

1

1

1

2

2

2

2

2

2

2

2

3

3

3

3

1

1

1

1

1

223

223

1

1

1

123

123

13

20

134

134

2

2

2

2

2

76

70

70

70

70

70

2

2

2

2

1

1

2

2

2

2

2

2

1

1

1

1

1

1

1

1
