# Supplementary figures and images for: Fungal Community Associated with Dactylopius (Hemiptera: Coccoidea: Dactylopiidae) and Its Role in Uric Acid Metabolism
Source: Front Microbiol. 2016 Jun 23;7:954. doi: 10.3389/fmicb.2016.00954 (PMC4917543; doi:10.3389/fmicb.2016.00954)

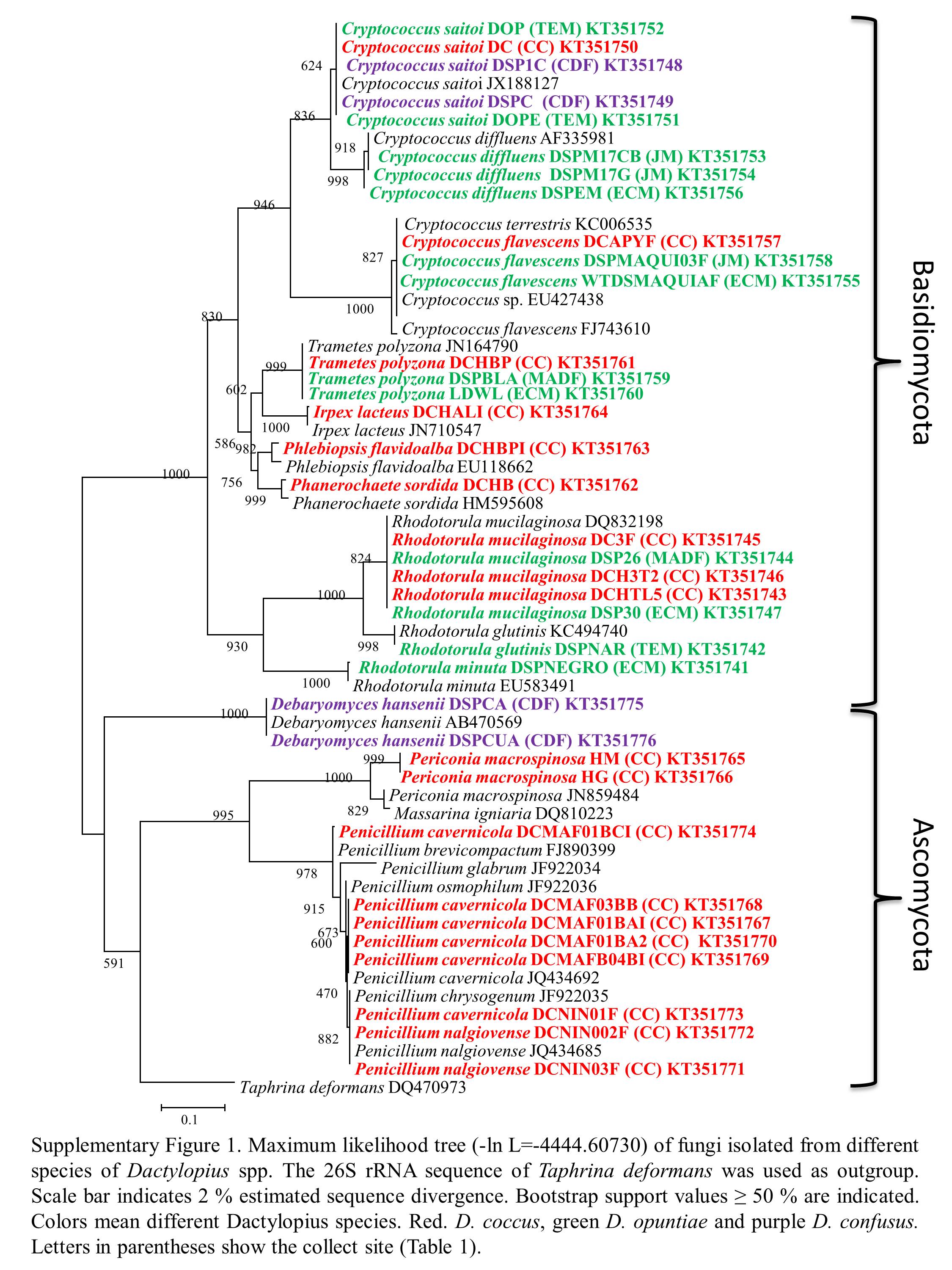

Supplement: Supplementary file 6 [file Image1.jpg]

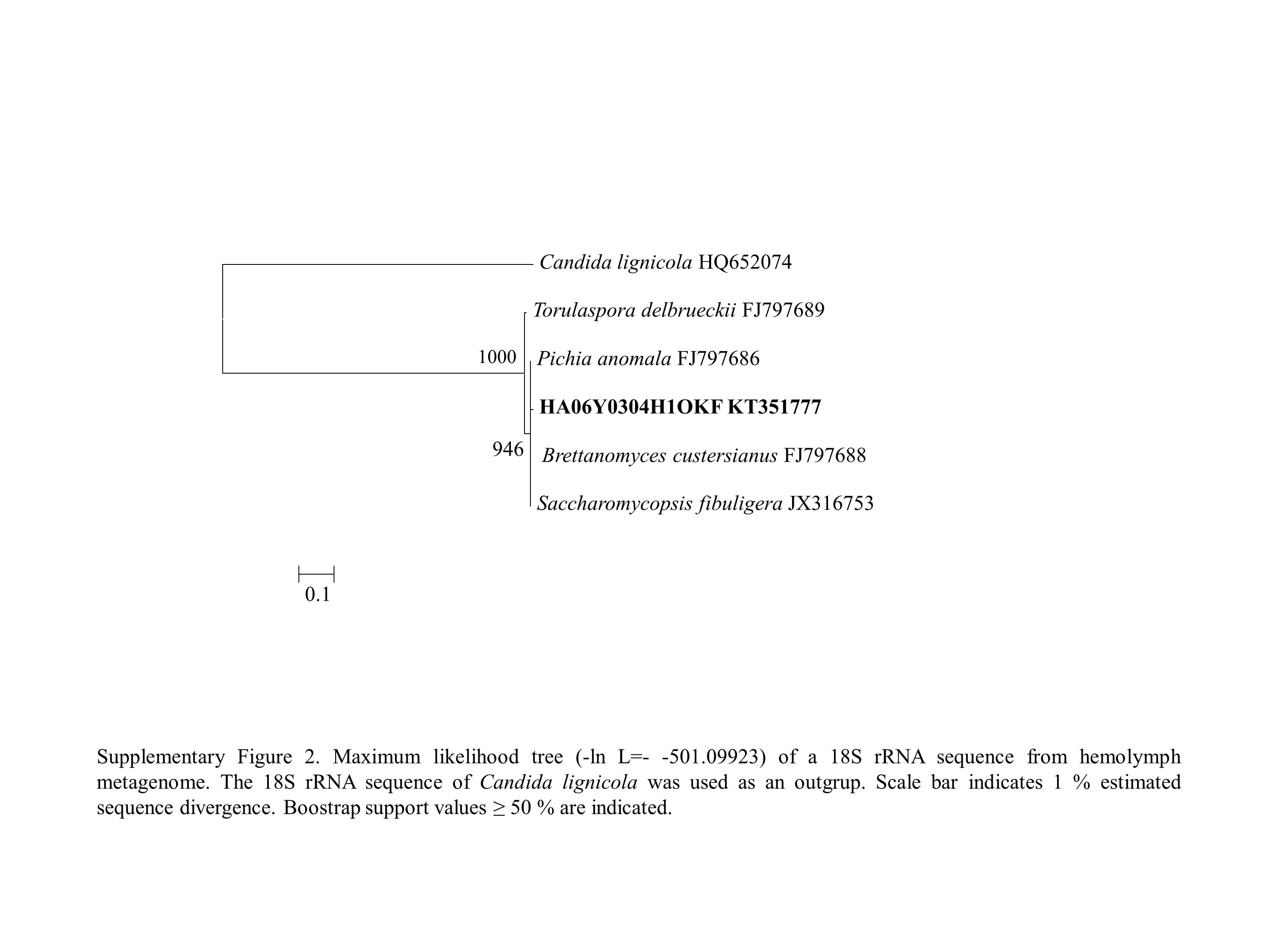

Supplement: Supplementary file 7 [file Image2.JPEG]

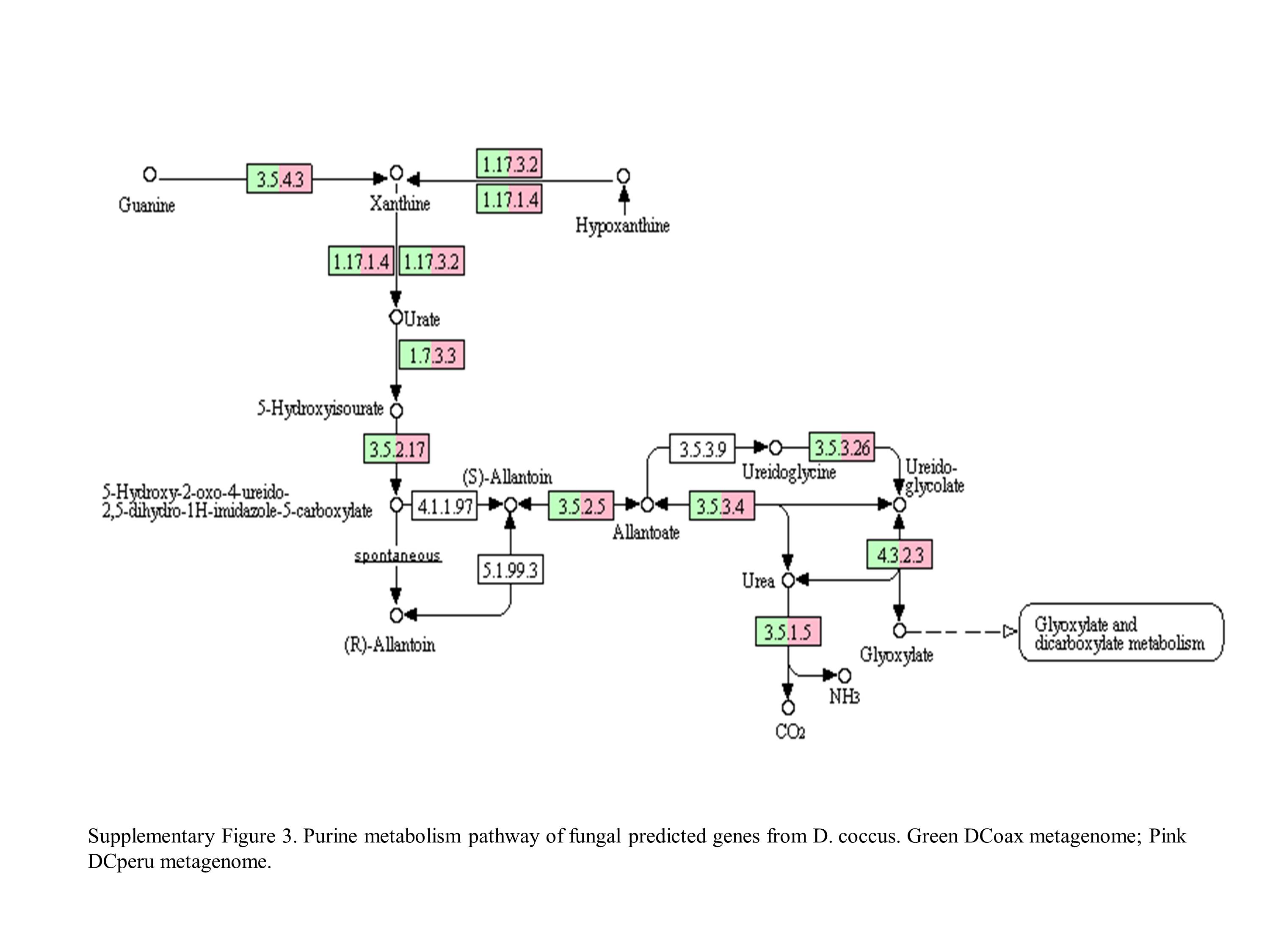

Supplement: Supplementary file 8 [file Image3.JPEG]

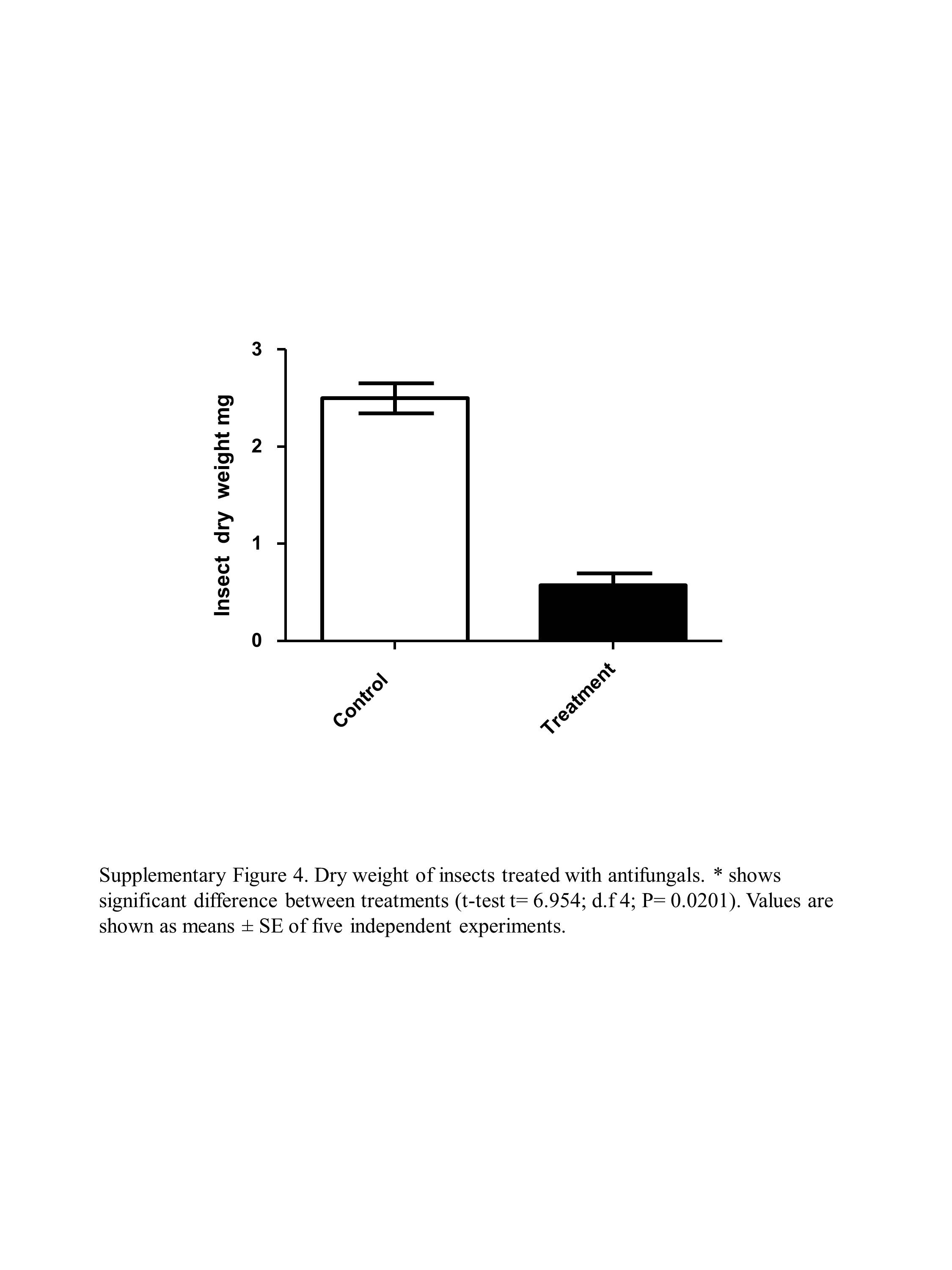

Supplement: Supplementary file 9 [file Image4.JPEG]

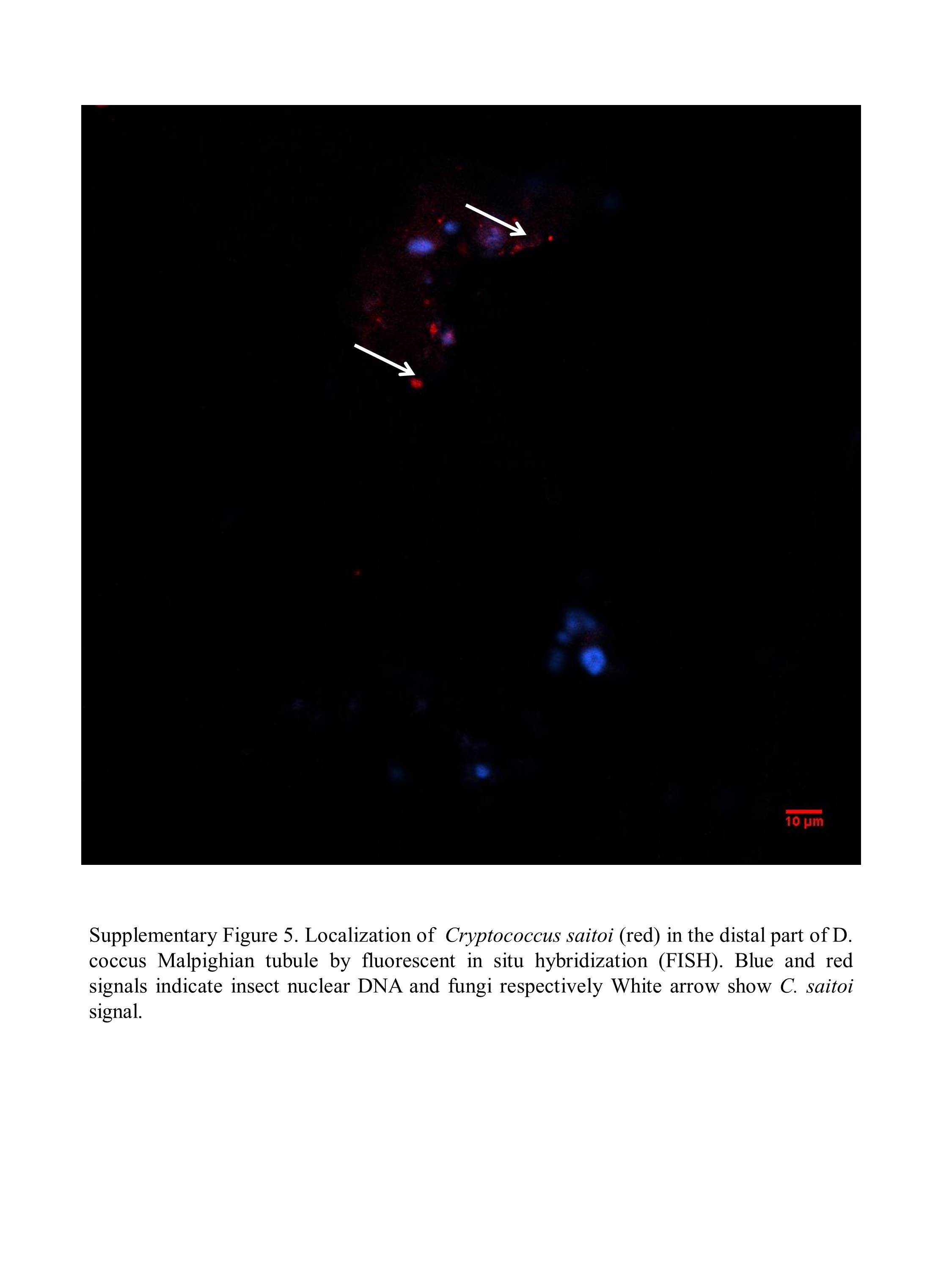

Supplement: Supplementary file 10 [file Image5.JPEG]
